# Depletion and Dysfunction of Dendritic Cells: Understanding SARS-CoV-2 Infection

**DOI:** 10.3389/fimmu.2022.843342

**Published:** 2022-02-21

**Authors:** Teding Chang, Jingzhi Yang, Hai Deng, Deng Chen, XiangPing Yang, Zhao-Hui Tang

**Affiliations:** ^1^Division of Trauma & Surgical Critical Care, Department of Surgery, Tongji Hospital, Tongji, China; ^2^Department of Immunology, Tongji Medical College, Huazhong University of Science and Technology, Wuhan, China

**Keywords:** COVID-19, SARS-CoV-2, dendritic cells, immunopathogenesis, severe acute respiratory syndrome coronavirus 2

## Abstract

Uncontrolled severe acute respiratory syndrome-coronavirus (SARS-CoV)-2 infection is closely related to disorders of the innate immune and delayed adaptive immune systems. Dendritic cells (DCs) “bridge” innate immunity and adaptive immunity. DCs have important roles in defending against SARS-CoV-2 infection. In this review, we summarize the latest research concerning the role of DCs in SARS-CoV-2 infection. We focus on the complex interplay between DCs and SARS-CoV-2: pyroptosis-induced activation; activation of the renin–angiotensin–aldosterone system; and activation of dendritic cell-specific intercellular adhesion molecule 3-grabbing non-integrin. We also discuss the decline in DC number, the impaired antigen-presentation capability, and the reduced production of type-I interferon of DCs in severe SARS-CoV-2 infection. In addition, we discuss the potential mechanisms for pathological activation of DCs to understand the pattern of SARS-CoV-2 infection. Lastly, we provide a brief overview of novel vaccination and immunotherapy strategies based on DC targeting to overcome SARS-CoV-2 infection.

## 1 Introduction

The coronavirus disease 2019 (COVID-19) pandemic poses a serious threat to public health and economic systems worldwide (1). As of January 27, 2022, more than 356.95 million people had been infected with the virus that causes COVID-19 [severe acute respiratory syndrome-coronavirus (SARS-CoV)-2] and 5.61 million individuals had died (data from WHO Coronavirus Dashboard).

Coronaviruses can cause intestinal and respiratory infections in animals and humans (2–6). SARS-CoV-2 is a genetically diverse virus found in a range of host species, including birds and mammals. It is transmitted mainly through the respiratory tract (7). The “spike” glycoprotein of SARS-CoV-2 binds to angiotensin-converting enzyme 2 (ACE2) and mediates membrane fusion and virus entry (8). SARS-CoV-2 infection induces pyroptosis (a highly inflammatory form of programmed cell death seen in cytopathic viruses), which leads to release of SARS-CoV-2, pathogen-associated molecular patterns (PAMPs), and damage-associated molecular patterns (DAMPs) (7, 9, 10). Innate immune cells are recruited by these products to respond to SARS-CoV-2 invasion and then release proinflammatory cytokines and “prime” the adaptive immune response *via* T cells and B cells. Due to unrestrained infiltration of inflammatory cells, the products induced by immune cells mediate lung injury by activating excess innate immune cells and oversecreting protease and reactive oxygen species (11). Therefore, understanding how innate immune cells respond to SARS-CoV-2 infection is critical for the prevention and treatment of COVID-19.

Dendritic cells (DCs) act as a “bridge” between innate immunity and adaptive immunity. They have important roles in viral infection. Pattern recognition receptors (PRRs) expressed on the membrane of DCs, such as Toll-like receptor (TLR)7, retinoic acid-inducible gene-I, melanoma differentiation-associated protein-5, and the cyclic guanosine monophosphate–adenosine monophosphate synthase–stimulator of interferon genes pathway, can recognize SARS-CoV-2-induced PAMPs and DAMPs (12–14). If these receptors are activated, a range of signaling pathways [e.g., interferon regulatory factor 3 (IRF3), IRF7, nuclear factor-kappa B (NF-κB)] are activated to regulate proinflammatory cytokines (e.g., tumor necrosis factor α (TNFα), interleukin (IL)-6, monocyte chemoattractant-1, macrophage inflammatory protein (MIP)1α, MIP1β) in the nucleus and induce interferon type I (IFN-I; the main cytokine responsible for producing a strong antiviral response) production (15, 16). DCs are also responsible for ingesting, transporting, processing, and presenting antigens to T cells, inducing the adaptive immune response, and have important roles in virus infection (17–19). However, the number of DCs, the ability to secrete antiviral cytokines (especially IFN-I), and the capability of antigen presentation decline unexpectedly in patients with severe COVID-19. This review focuses on explaining the (i) interrelationship between DCs and SARS-CoV-2 and (ii) reduced number and dysfunction of DCs. In this way, we hope to create a breakthrough in immunomodulation therapy and an IFN strategy against COVID-19.

## 2 Direct and Indirect Interaction of SARS-CoV-2 With DCs

### 2.1 Pyroptosis: A Major Inducer for SARS-CoV-2 to Attract DCs

There is growing evidence of cytolysis (mostly pyroptosis but not necroptosis) in patients infected with SARS-CoV-2 (7, 20). “Pyroptosis” refers to the biological process that relies on caspase-1-dependent activation of gasdermin D to form membrane pores. “Necroptosis” is dependent upon the intracellular signal transduction of receptor-interacting protein kinases, and membrane pores are formed through phosphorylation of mixed lineage kinase domain-like pseudokinase, which results in cytolysis (21). *In vitro* studies have shown that caspase-1 is activated in patients with severe COVID-19, downstream secretion of IL-1β increased, and decomposition of gasdermin D accelerated (22). Those data show that cytolysis during SARS-CoV-2 infection is mainly pyroptosis rather than necroptosis. Nucleotide-binding oligomerization domain-like receptors (NLRs) recognize the danger signals (which are derived from homologous hosts or microorganisms) and form large supramolecular chemical inflammasomes (9). Caspase-1 activation may be related to SARS-CoV-2 recognition by NLRs in cells. After caspase-1 has been activated, it initiates the pyroptosis pathway, activates gasdermin D to form plasma-membrane pores, and allows water influx, cell swelling, and osmotic lysis. Activated caspase-1 can also activate IL-1β and IL-18, which are released mainly through plasma-membrane pores (9). Many PAMPs, DAMPs, and virus products, which are released by SARS-CoV-2-induced pyroptosis, recruit various types of immune cells (including DCs) to infiltrate lung tissue and promote secretion of cytokines, particularly IL-6, IL-1β, IL-10, TNF, granulocyte macrophage-colony stimulating factor, IFN-induced protein-10, IL-17, monocyte chemoattractant-3, and IL-1ra (23).

ACE2 is the main target of SARS-CoV-2 (11). We postulate that ACE2 is also the inducer of pyroptosis caused by SARS-CoV-2. ACE2 is a transmembrane protease and is the main receptor for virus invasion (24). The N-terminal extracellular domain of ACE2 comprises a “claw-like” protease domain (PD). The receptor-binding domain (RBD) of SARS-CoV-2 combines with the PD of ACE2 to form an RBD–PD complex (24). The C-terminus is a transmembrane domain, also known as the collectrin-like domain (24). Studies have suggested that ACE2 is the major receptor of SARS-CoV and SARS-CoV-2 and binds to transmembrane serine protease (TMPRSS)2-activated spike proteins to induce virus entry into endosomes (25). ACE2 and TMPRSS2 are expressed primarily in cells of the upper respiratory tract and lungs (11, 25–27). These respiratory-tract cells with special cell-membrane receptors are ideal targets for SARS-CoV-2 invasion. This phenomenon may explain why SARS-CoV-2 is transmitted through aerosols and is very infectious (7).

The increased levels of lactate dehydrogenase (LDH) (28) and D-dimer in the plasma of patients infected with SARS-CoV-2 suggest that there is a high level of tissue injury in patients with severe COVID-19 (22, 23, 29, 30). Patients with severe COVID-19 with high LDH levels and leukopenia have impaired integrity of cell membranes (23, 31–33). In addition, the full blood count and biochemical findings of patients with severe COVID-19 reveal that the leukopenia observed in such patients appears to precede the “cytokine storm” (31, 34). SARS-CoV-2 infects human primary monocytes *in vitro*, resulting in their lysis and death. Flow cytometry and fluorescence microscopy have demonstrated the membrane disruption triggered by SARS-CoV-2 (22). In conclusion, we believe that SARS-CoV-2 invades cells through the ACE2, induces pyroptosis, and finally, causes the subsequent cytokine storm, leading to disease progression.

**Figure 1:**
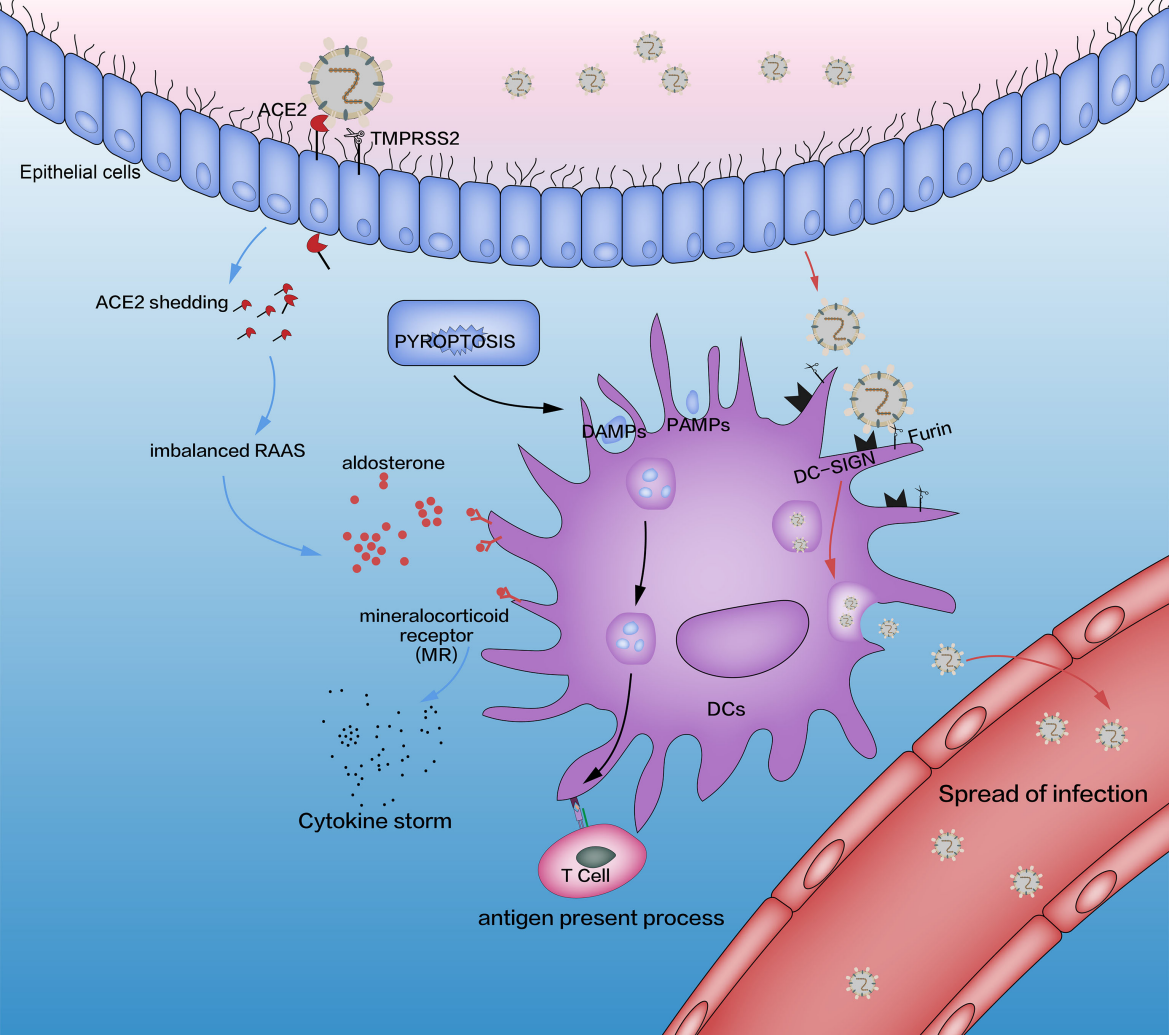
Interaction between SARS-CoV-2 and DCs (schematic). The three pathways affected by SARS-CoV-2 are pyroptosis (black lines), imbalanced RAAS (blue lines), and DC-SIGN (red lines). The adaptive immune system is induced by pyroptosis, which is activated by SARS-CoV-2. In addition, SARS-CoV-2 combines with the ACE2 receptor through its spike protein (S), which is activated by TMPRSS2. This process causes an imbalance in the RAAS through the shedding of ACE2 and releases excessive amounts of aldosterone, which promotes the release of proinflammatory cytokines in DCs through MRs. SARS-CoV-2 impacts DCs directly by DC-SIGN, a receptor which has critical roles in the recognition of viruses (e.g., HIV, Ebola, dengue, cytomegalovirus) and other pathogens (e.g., Leishmania species, *Candida albicans*, *Mycobacterium tuberculosis*, *Streptococcus pneumoniae*, *Aspergillus fumigatus*). Although SARS-CoV-2 replication in lung cells is well-documented, a similar process has not been confirmed in alveolar DCs. Some researchers have suggested such a replication based on triggering aberrant production of proinflammatory cytokines/chemokines and inducing the spread of SARS-CoV-2 infection, as is the case with SARS-CoV and MERS-CoV, but other scholars have ruled out SARS-CoV replication in human DCs. RAAS, renin–angiotensin–aldosterone system; DCs, dendritic cells; TMPRSS2, transmembrane serine protease 2; MRs, mineralocorticoid receptors; DC-SIGN, dendritic cell-specific intercellular adhesion molecule 3-grabbing non-integrin.

Products which are induced by SARS-CoV-2 attract DCs selectively into inflammatory sites (35–38). DC aggregation has been observed in the bronchoalveolar lavage fluid of COVID-19 patients, which suggests that DCs infiltrate into lung tissues by the products of SARS-CoV-2-induced pyroptosis (35–38) (**Figure 1**). Flow cytometry of the bronchoalveolar lavage fluid of COVID-19 patients has revealed that only type-2 conventional dendritic cells (cDC2) accumulate in the lungs (35, 36). cDC1 and plasmacytoid dendritic cells (pDCs), which are involved in IFN-I secretion, are absent in infected lungs (35, 36). cDC2 support the cluster of differentiation (CD)4+ T-cell response, stimulate follicular T-helper (Th) cells (which activate a humoral antiviral adaptive immune response), and produce inefficient antiviral proinflammatory cytokines (39–41).

SARS-CoV-2 attacks nasal cells and lung cells through ACE2, activates pyroptosis, and leads to the production of many DAMPs, PAMPs, and progeny viruses. These products activate innate immunity (**Figure 1**).

### 2.2 Imbalanced Renin–Angiotensin–Aldosterone System: Potential Regulation of DCs

SARS-CoV-2 enters cells through the ACE2. ACE2 is a key factor in the renin–angiotensin–aldosterone system (RAAS) (42, 43). SARS-CoV-2 may affect the RAAS and lead to disease development by affecting the shedding of ACE2. The RAAS mediates blood-pressure control, inflammation, sodium reabsorption, and fibrosis (44). RAAS disorders can lead to heart failure, low blood pressure, atherosclerosis, and diabetes mellitus (45). The factor related most closely with ACE2 in the RAAS is angiotensin (Ang)II. The latter stimulates vascular contraction, secondary inflammation, and atherosclerosis through the type-1 angiotensin II receptor (AT1R) (46). Another receptor, type-2 angiotensin II (AT2R), in contrast to AT1R, is activated by AngII to promote vascular dilation, platelet aggregation, and promotion of insulin action. However, AT2R is rarely expressed in healthy adults (47). Therefore, the regulation and balance of AngII are dependent mainly on ACE2. The latter can convert AngII to Ang-(1–7), which is similar to AT2R stimulation (44, 47). An excess of AngII promotes pulmonary vascular contraction, inflammation, and cytokine-induced organ damage, increases the permeability of cell membranes and apoptosis, and induces acute kidney injury and acute respiratory distress syndrome (ARDS). AngII overactivation is associated with an increase in ACE2 shedding in patients with COVID-19. In a cohort of 12 COVID-19 patients, the circulating level of AngII was significantly higher than that in healthy controls (linearly correlated with viral load), which suggested a direct link between RAAS imbalance and multiorgan damage caused by SARS-CoV-2 infection (1, 48, 49).

Among hospitalized COVID-19 patients, the increase in AngII level is accompanied by an increased level of IL-6, with the highest mortality rate (29). This phenomenon has also been found in the patients with avian influenza A (H7N9) infection. Within 4 weeks of H7N9 infection, AngII levels increase gradually, which is associated with a worse clinical prognosis (50). In COVID-19 patients, AngII levels have been found to be closely related to viral titer and partial pressure of oxygen in arterial blood/fraction of inspired oxygen (49). COVID-19-induced AngII aggregation has been shown to promote acute lung injury (ALI) by activating cytokine-induced inflammation, activating the nicotinamide adenine dinucleotide hydrogen (NADH)/nicotinamide adenine dinucleotide phosphate hydrogen (NADPH) oxidation system and vasoconstriction (49, 51, 52). Researchers found that COVID-19 was more severe and carried a worse prognosis in older people and males, if ACE2 expression declined (49, 53, 54). Coincidentally, patients with COVID-19 with diseases associated with RAAS overactivation (e.g., hypertension, diabetes mellitus) have been shown to carry a higher risk of transfer to the intensive care unit and mortality (55). RAAS overactivation appears to be closely related to a poor prognosis in COVID-19 patients, and the loss of ACE2 (which can regulate the effects of an overactive RAAS during the disease) will lead to worse consequences.

The ACE expressed on the DCs is still able to participate in the RAAS disorders (56–58). It has been proved that the ACE expression increased was correlated with the differentiation and stimulation of DCs (59). Elevated ACE is involved in the production of AngII and the peptide repertoire trimming as part of the MHC-II complex (59, 60). Accumulation of AngII due to the shedding of ACE2 leads to the phosphorylation of the ERK and promotes the secretion of IL-6 and TNFα (61). In the meantime, AngII has the ability to improve the migration, maturation, and antigen presentation of DCs to improve Th1 cells (62).

The increase in the AngII level is accompanied by an increase in the aldosterone level because AngII can stimulate the adrenal cortex to secrete aldosterone. DCs are key immune cells involved in severe COVID-19-induced lung damage mediated by aldosterone, which can stimulate DCs to produce IL-6 and transforming growth factor-β1 *via* the mineralocorticoid receptor (**Figure 1**) (63, 64). AngII and aldosterone can alter the proliferation and maturation of DCs, leading to DC dysfunction (36). Supplementation of ACE2 may remedy this problem. Recombinant human (rh)ACE2 has a protective effect in SARS-CoV-induced ALI, and injection of rnACE2 can reduce inflammation and improve lung function (65–67). Therefore, rhACE2 may be a potential treatment for SARS-CoV-2-induced ALI.

### 2.3 Dendritic Cell-Specific Intercellular Adhesion Molecule 3-Grabbing Non-Integrin: Direct Interaction Between SARS-CoV-2 and DCs

Yang and colleagues (68) showed that SARS-CoV-2 enters DCs and macrophages through dendritic cell-specific intercellular adhesion molecule 3-grabbing non-integrin (DC-SIGN) and furin rather than through ACE2 and TMPRSS2. DC-SIGN (CD209) is a C-type calcium-dependent lectin and a type-II membrane protein. DC-SIGN consists of three domains: extracellular, transmembrane, and intracellular (69–72). The intracellular domain is an N-terminal domain responsible for the binding, phagocytosis, and intracellular transport of molecules associated with signal transduction. The transmembrane domain anchors DC-SIGN to the cell membrane. The extracellular domain consists of two portions: a neck domain (which forms a tetramer that stabilizes the extracellular part of the molecule) and a c-type carbohydrate-recognition domain (a calcium-dependent receptor with a highly conserved sequence) (43, 69, 73). The extracellular domain is essential for binding and recognizing high-mannose oligosaccharides (43, 74, 75). DC-SIGN is expressed exclusively by mature and immature DCs in the skin, mucosa, and lymphoid organs (76). It is a PRR/adhesion receptor in DCs that promotes migration and adhesion and mediates the inflammatory response by activating innate immune and adaptive immune systems. Remarkably, DC-SIGN has a pivotal role in the “immune escape” of pathogens and tumor cells (70, 71). As an antigen-capture receptor, DC-SIGN recognizes, internalizes, and decomposes antigens and, eventually, presents them to CD4+ T cells to trigger an immune response (77). However, virus-infected DCs, through DC-SIGN, also spread SARS-CoV-2 to other tissues and organs (**Figure 1**). This phenomenon is typical in human immunodeficiency virus (HIV) infection, where the DC-SIGN signal is activated by the HIV and induces its migration to lymph nodes. In lymph nodes, DC-SIGN promotes the migration of HIV-1 from DCs to CD4+ T cells, thereby promoting cell infection and virus transmission (78–80). Hence, whether this “Trojan horse” phenomenon exists in SARS-CoV-2 infection is worthy of further study.

The role of DC-SIGN in SARS-CoV-2 infection is not known. Recent studies have shown that SARS-CoV binds DC-SIGN through its activated spike protein and takes part in the inflammatory response of DCs (**Figure 1**) (43, 81, 82). DC-SIGN also plays an important part in Middle East respiratory syndrome-related coronavirus (MERS-CoV) infection and is too crucial to neglect in SARS-CoV-2 infection (83). DC-SIGN could become an alternative receptor for SARS-CoV-2 to invade DCs. It has been suggested that the interaction of SARS-CoV-2 or its envelope proteins (including the products of pyroptosis) with DC-SIGN result in the loss of a specific DC subpopulation (36). Moreover, activated DC-SIGN receptors downregulate the expression of major histocompatibility class (MHC)-II molecules (84), which is a key factor of impaired presentation of antigens. In addition, the combination between DC-SIGN and HIV can promote a Th2 cell-based immune response, thereby reducing levels of IL-12 and IFNs (42), which helps to explain the innate immunosuppression in patients with severe COVID-19.

The DC-SIGNR (L-SIGN, CD209L), which is the homology receptor of the DC-SIGN, is broadly expressed in both endothelial cells and epithelial cells (85, 86). The DC-SIGN/L-SIGN may be an alternative receptor, due to the fact that ACE2-deficient endothelial cells but not DC-SIGNR-deficient endothelial cells allow SARS-CoV-2 entry (85, 86). Michel et al. confirm the important role of DC-SIGN in the Trojan Horse model of DCs during SARS-CoV-2 infection (87). They think that SARS-CoV-2 adheres to the surface of DCs through DC-SIGN and secondly present SARS-CoV-2 to susceptible cells in the process recognized as trans-infection which relies on the characteristics of DCs’ migration (87). Intriguingly, the DC-SIGN gene expression is interestingly decreased in lung DCs but increased in circulation DCs which would undoubtedly increase the amount of SARS-CoV-2 carried by DCs and enhance its global transmission ability (88).

As in HIV infection, DC-SIGN-mediated virus internalization is a critical mechanism for immune escape in SARS-CoV-2 infection (75). DC-SIGN has become another potential invasion portal for SARS-CoV-2, and its function deserves further exploration in COVID-19 patients. However, after SARS-CoV-2 infection, the depletion and dysfunction of DCs result in persistent virus infection. The exact mechanism by which SARS-CoV-2 infection reduces the number and function of DCs is discussed below.

## 3 Depletion and Dysfunction of DCs in COVID-19

### 3.1 Decline in the Number of DCs in COVID-19 Patients

cDC1, cDC2, and pDCs are recruited into lung tissue during infection (39). cDC1s (also known as CD141+ DC) are found in peripheral blood and among resident DCs of the lymph nodes, bone marrow, and spleen (39, 89–93). They participate in cross-presentation of antigens *via* MHC-I molecules to activate CD8+ T cells and promote Th1 cells and natural-killer-cell responses though IL-12 (39). cDC1s also secrete IFN-III (including IFN-λ) (39, 94). cDC2s (also known as CD1c+ DC) are present mainly in peripheral blood, lymphoid organs, and tissues. They are activated to become robust producers of IL-12 and are excellent cross-presentation cells. cDC2s are also major producers of IL-23, IL-1, TNF-α, IL-8, and IL-10 (39, 95–97). pDCs (which were identified first in peripheral blood and tonsils) “sense” and respond to viral infection through rapid production of IFN-I (39, 98, 99). These three types of DCs are activated by PAMPs and DAMPs to promote the innate immune response and to activate the adaptive immune response.

**Figure 2:**
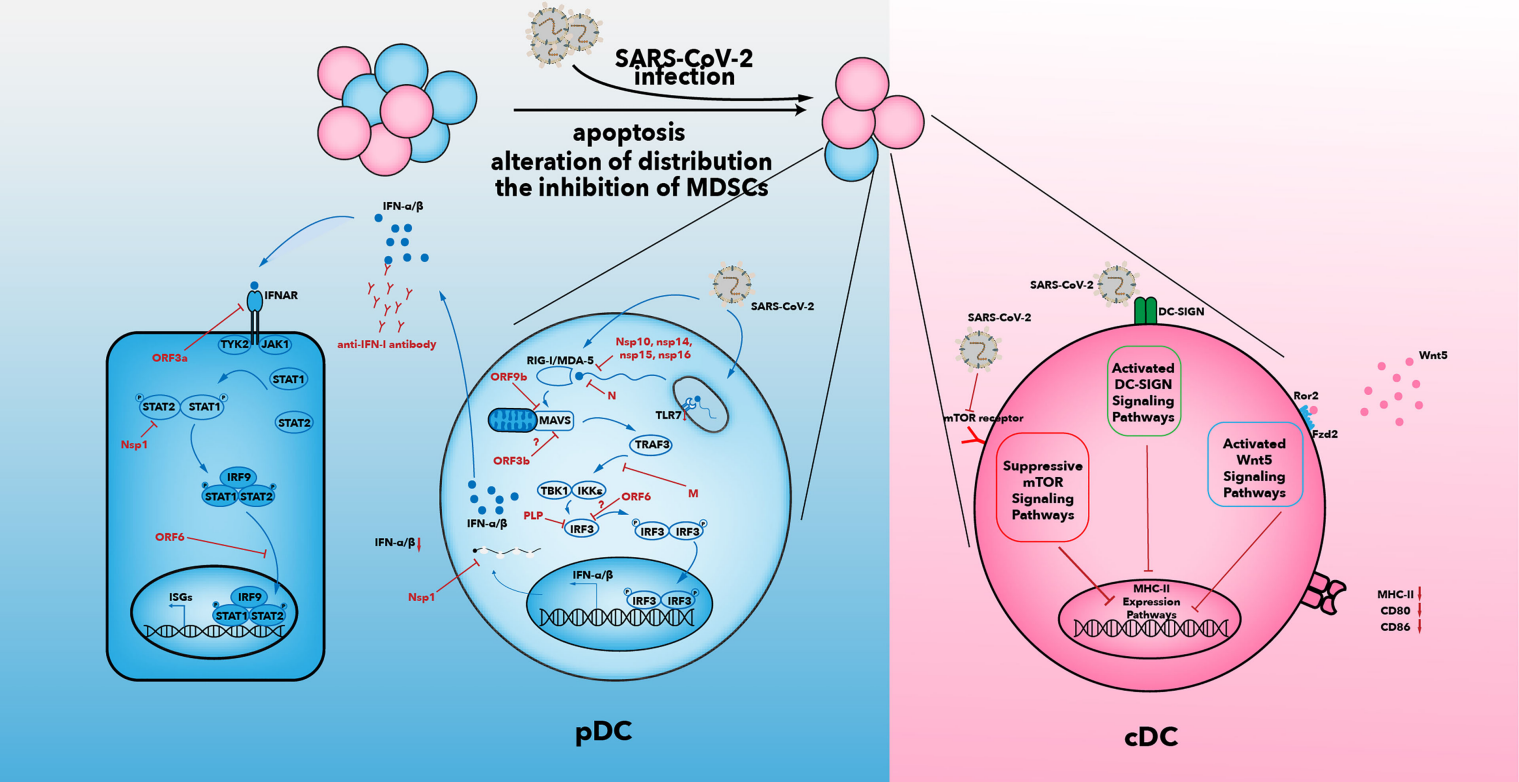
Multiple suppressive mechanisms in SARS-CoV-2-infected DCs from patients with severe COVID-19. The number of DCs in patients decreases after SARS-CoV-2 infection. Increased apoptosis, alterations in distribution of DCs, and inhibition of MDSCs may be associated with a decrease in DC number. IFN-I secretion is inhibited by various viral proteins that have been shown to be effective against IFN-I signaling in SARS-CoV infection (104). In addition to the effects of viral proteins, anti-IFN antibody and reduced expression of TLR7 have been observed in some patients with severe COVID-19. The capability of antigen presentation is impaired in cDC1 and cDC2. Inhibition of the mTOR signaling pathway, activated DC-SIGN pathway, and activated Wnt5 pathway could contribute to downregulation of MHC-II and co-stimulatory molecules. IFN, interferon; IFNAR, interferon alpha and beta receptor; mTOR, mammalian target of rapamycin; IκB, inhibitor of nuclear factor κB; IRF, IFN regulatory factor; ISG, IFN-stimulated gene; JAK, Janus kinase; IKKϵ, IκB kinase-ϵ; M, membrane; MAVs, mitochondrial antiviral signaling proteins; N, nucleocapsid; Nsp, non-structural protein; ORF, open reading frame; P, phosphate; DC-SIGN, dendritic cell-specific intercellular adhesion molecule 3-grabbing non-integrin; PLP, papain-like protease; RIG-I, retinoic acid-inducible gene-I; MDA5, melanoma differentiation-associated gene 5; SARS-CoV-2, severe acute respiratory syndrome-coronavirus 2; TANK, TRAF family member-associated NF-κB activator; TBK1, TANK-binding kinase 1; TRAF3, tumor necrosis factor receptor-associated factor 3; STAT, signal transducer and activator of transcription; TYK2, tyrosine kinase 2; Wnt5, Wnt oncogene analog 5.

The number of DCs in the blood of COVID-19 patients is reduced. Among the three types of DCs, only cDC2s accumulate in the lungs of COVID-19 patients (35, 100–103). The mechanisms that result in a decline in the number of DCs may be caused by an alteration in distribution of DCs, increased apoptosis of DCs, and the inhibitory effects of myeloid-derived suppressor cells (MDSCs) (**Figure 2**).

#### 3.1.1 Alteration in the Distribution of DCs

SARS-CoV-2 causes pyroptosis through infection of respiratory epithelial cells *via* ACE2 and TMPRSS2 (11, 27) and leads to production of plentiful cytokines and chemokines that attract DCs to migrate from peripheral blood into the lungs (7, 20, 21). Sanchez-Cerrillo and coworkers (35) analyzed DC profiles in the blood and lungs of COVID-19 patients. They found that pDCs and cDC1s showed a significant depletion and that cDC2s migrated from the blood to the lungs. Xiong and colleagues (38) observed abundant mature DCs in bronchoalveolar lavage fluid, which indicated DC accumulation in SARS-CoV-2-infected lungs. Insufficient recruitment of pDCs and cDC1s to the lungs may be due to downregulation of chemokine receptors such as C–C chemokine receptor type-2 and C–X–C motif chemokine receptor-3 (100). A reduction in the circulation of pDCs and cDC1 may be a result of migration to or sequestration in lymphoid tissues (105, 106). Changes in the distribution of cDC2 may contribute to the reduction in circulation of DCs.

#### 3.1.2 Incremental Apoptosis of DCs

The circulation of DCs (especially pDCs) is diminished significantly in COVID-19 (35). pDCs are key factors involved in the antiviral efficacy of IFN-I (39). Hence, SARS-CoV-2 may target pDCs and reduce their number. Saichi and colleagues found p53 signaling to be upregulated in pDCs in COVID-19 cases (105). However, Onodi and colleagues found that pDCs, which were isolated from healthy donors, are improved during SARS-CoV-2 infection (107), thereby suggesting that SARS-CoV-2 could not kill pDCs directly. In sum, other mechanisms must be involved in pDC apoptosis.

Recently, “immunometabolism reprogramming” has been postulated to explain why DC apoptosis is increased during SARS-CoV-2 infection (108). Immunometabolism reprogramming is characterized by changes in the metabolic stages of immune cells from homeostasis to an inflammatory environment or infectious environment (108). Recent studies have revealed that DC apoptosis in COVID-19 patients was because of the TNF-related apoptosis-inducing ligand or apolipoprotein-2 ligand but was not dependent on the Fas ligand (108–111). The displacement of hexokinase-II from mitochondrial-associated membranes increases glycolysis to produce glucose-6-phosphate. This action can lead to excess release of Ca^2+^ from the endoplasmic reticulum to the cytosol to activate calpains, mitochondrial depolarization, and apoptosis (112).

#### 3.1.3 Inhibitory Effect of MDSCs

MDSCs are induced in pathological conditions such as inflammation, cancer, or some autoimmune disorders. They inhibit immature myeloid cells from differentiating into mature DCs, upregulate expression of immunosuppressive factors (e.g., inducible nitric oxide synthase, arginase), and promote the production of nitric oxide and reactive oxygen species. Those productions result in expansion of an immunosuppressive population of immature myeloid cells and lead to DC depletion (113, 114). Recent studies have found that the increased number of MDSCs in COVID-19 patients might be key to the decline in DC number (101, 115).

### 3.2 Impaired Antigen Presentation of SARS-CoV-2-Infected DCs

Not only does the number of DCs decline, but also the ability of DCs to present antigens is impaired during SARS-CoV-2 infection (101, 105, 115, 116). Zhou et al. studied the antigen-presentation capacity of circulating DCs in the acute phase of COVID-19. They found that expression of the co-stimulatory molecule (CD80, CD86) and antigen-presentation molecule (human leukocyte antigen-DR) decreased after stimulation with a “cytokine cocktail” (IL-1β, IL-6, TNF-α, prostaglandin E2) (101). DCs in the acute phase of COVID-19 failed to stimulate the proliferation of CD4+ and CD8+ T cells according to a mixed lymphocyte reaction assay with allogeneic CD4+ and CD8+ T cells (101). The antigen-presentation capability of DCs could act as a bridge between innate immunity and adaptive immunity. Determining the reason for the decline in the antigen-presentation capacity of DCs is crucial, and some reasons are discussed in the following (**Figure 2**).

#### 3.2.1 Impaired Mammalian Target of Rapamycin Signaling

Impaired mammalian target of rapamycin (mTOR) is a crucial regulator of the development, maturation, and function of DCs. mTOR can dictate and “shape” the inflammatory immune response of DCs (117). Inhibition of mTOR expression hinders DC maturation and reduces the antigen uptake in the early stage of infection (117, 118). Prabhu et al. found a decrease in the pS6 (mTOR marker) level in blood, thereby indicating inhibition of the mTOR signal in COVID-19 patients (115). Suppression of the mTOR signaling pathway may contribute to the impaired antigen presentation of DCs in COVID-19 patients.

#### 3.2.2 Upregulation of Wnt Oncogene Analog Expression

Wnt oncogene analog (Wnt)5 can impair the function and maturation of DCs (104, 119). Wnt5a is activated in ARDS and sepsis as an inhibitor of the repair process (119). Wnt5a could be a candidate biomarker of SARS-CoV-2 progression (116, 120). The Wnt5 signaling pathway may also impair the function of DCs to present antigens to T cells.

#### 3.2.3 Summary

The mechanism by which the antigen-presentation capability of DCs is impaired is not clear. A greater focus on the SARS-CoV-2-induced microenvironment (including alteration of signaling pathways such as DC-SIGN, mTOR, and Wnt5) may be needed to ascertain the underlying mechanism.

### 3.3 IFN-I Deficiency in SARS-CoV-2-Infected DCs

IFN-I is a powerful weapon for DCs in their fight against viral infection. However, SARS-CoV-2 often leads to the effects of IFN-I being hampered. Zhou et al. found that the ability of infected cDC1 and cDC2 to produce proinflammatory cytokines (IL-1β, IL-6, TNF-α) was not influenced, but the ability of infected pDCs to secrete IFN-I was suppressed significantly, in COVID-19 patients (101). Achille et al. found that the ability of cDC1 to produce IFN-λ was not impaired, which may contribute to disruption of the epithelial barrier in the lungs upon SARS-CoV-2 recognition (121). Various studies have shown that SARS-CoV-2 inhibits only pDCs from secreting IFN-I but does not affect cDC production of other proinflammatory cytokines (68, 101, 115, 116). An IFN-I deficiency promotes SARS-CoV-2 to escape recognition by the immune system. Such an escape by SARS-CoV-2 leads to the body producing many inefficient antiviral proinflammatory cytokines that will initiate the cytokine storm that damages lung tissues and results in ARDS or death. Three main reasons have been postulated as to how SARS-CoV-2 may specifically impair the production and transformation of IFN-I (**Figure 2**).

First, the IFN-I level is reduced significantly in coronavirus infections (e.g., SARS-CoV, MERS-CoV) and such inhibition is a hallmark of coronavirus infections (122–124). Little is known about how SARS-CoV-2 suppresses IFN signaling, but clues can be obtained from SARS-CoV. IFN-I production in SARS-CoV-2 infection is even less than that observed in SARS-CoV infection (124). SARS-CoV-2 encodes various proteins that have been shown to inhibit IFN-I signaling in SARS-CoV infection (122, 124) (**Figure 2**).

Second, some researchers have found congenital deletion of TLR7 in critically ill patients and that the pDCs of patients with a TLR7 deletion cannot produce IFN-I (125). In addition, anti-IFN antibodies have been found in a small number of critically ill patients; anti-IFN antibodies bind to IFN and prevent IFN transformation (126). The failure of the immunometabolism reprogramming such as the impairment of the glycogenolysis-mediated glycolysis which is stored as a source of energy of DCs will impair the IFN-I generation in DCs (108, 127, 128). SARS-CoV-2 inhibits the early induction of glycolysis *via* suppression of IFN-I generation, and in turn, the impaired induction of glycolysis inhibits IFN-I generation in DCs (129). This positive feedback loop further reduces IFN-I generation.

Third, the high viremia of COVID-19 is associated with impaired generation of IFN-I in peripheral blood mononuclear cells (130). Supplementation of IFN in the early stage of SARS-CoV-2 infection is beneficial to patients. IFN-β combined with lopinavir and ribavirin can improve physical status (131) and alter the production of proinflammatory cytokines in the latter stages of COVID-19 (97, 132). There is growing recognition that inappropriate, excessive, and mistimed IFN treatments are deleterious in viral infections (131, 133). In the early stage of COVID-19, IFN treatment could improve patient outcomes (122, 131, 134–136), but immunomodulation therapy is recommended at the latter stage of COVID-19 (122, 137, 138). The delayed response to IFN-I leads to accumulation of pathogenic mononuclear macrophages, which cause vascular leakage, lung disorders, and inappropriate T-cell responses (139, 140). Understanding the exact immunopathogenesis of impaired IFN production and restoring the IFN system at the early stage of COVID-19 is a top priority.

## 4 Conclusions

As a bridge between innate immunity and adaptive immunity, DCs have important roles during virus invasion. The impaired function and reduced numbers of DCs are a catastrophe for the immune system during SARS-CoV-2 infection. The deficiency and dysfunction of DCs persist for several months after SARS-CoV-2 infection (113). Seven months after SARS-CoV-2 infection, the function of cDC2, as well as the number and IFNα production in pDCs, remains abnormal (113). This prolonged deficiency and dysfunction of DCs are associated with “post-acute COVID-19 syndrome” (“long-hauler syndrome”) in COVID-19 patients. It is characterized by persistent symptoms and/or delayed or long-term complications of SARS-CoV-2 infection beyond 4 weeks from symptom onset (141, 142). Scholars have reported that SARS-CoV-2 can persist in the intestines 7 months after symptom resolution (143). We postulate that the persistent tissue damage and presence of viral antigens which are hard to eliminate due to the deficiency and dysfunction of DCs may contribute to long-hauler syndrome in COVID-19 patients.

The morbidity and mortality of older patients are very high if they have severe SARS-CoV-2 infection (144). Age-associated DC dysfunction has a critical role in the mortality prevalence of COVID-19 patients (103). Age may contribute to the reduction and IFN dysfunction of pDCs and antigen-presentation inhibition of cDC to CD8+ T cells, which hinders the transition from naïve CD8+ T cells to cytotoxic CD8+ T cells (145). In older patients, DC dysfunction leads to an increased proinflammatory response and decreased anti-inflammatory and immunomodulatory responses, which result in a chronic inflammatory state (103). In SARS-CoV-2 infection, DC dysfunction could lead to uncontrolled infection and exacerbate the cytokine storm in older patients (103).

As important antigen-presenting cells, DCs have a critical role in the immunotherapy of SARS-CoV-2. Development of immunotherapies against SARS-CoV-2 includes classic platforms and next-generation platforms. The ongoing vaccine research on classic platforms includes whole-inactive viruses, live-attenuated viruses, protein subunits, and virus-like particles. Next-generation platforms include viral vectors, DNA, RNA, and antigen-presenting cells. LV-SMENP-DC and pathogen-specific artificial antigen-presenting cells (aAPC) from Shenzhen Genoimmune Medical Institute (Shenzhen, China) have recently moved into phase-I clinical development. LV-SMENP-DC is an antigen-presenting-cell vaccine based on DCs. In LV-SMENP-DC, DCs are modified with a lentiviral vector expressing a “synthetic minigene” based on the domains of selected viral proteins, and LV-SMENP-DC is administered with antigen-specific cytotoxic T-cells (NCT04276896) (146, 147).

This review shows that DCs have vital roles in SARS-CoV-2 infection. Studying the relationship between DCs and SARS-CoV-2 is very important. Novel DC-induced immunotherapy strategies for COVID-19 may be discovered in the near future.

## Author Contributions

T-DC and Z-HT participated in the design and drafting of the manuscript. JZY, HD, and DC participated in critical discussions and revised the manuscript. TC prepared the figures. Z-HT supervised the project. All authors contributed to the article and approved the submitted version.

## Funding

This work was supported in part by the National Natural Science Foundation of China (81873870).

## Conflict of Interest

The authors declare that the research was conducted in the absence of any commercial or financial relationships that could be construed as a potential conflict of interest.

## Publisher’s Note

All claims expressed in this article are solely those of the authors and do not necessarily represent those of their affiliated organizations, or those of the publisher, the editors and the reviewers. Any product that may be evaluated in this article, or claim that may be made by its manufacturer, is not guaranteed or endorsed by the publisher.
